# Preface to special topic on brain–machine interface

**DOI:** 10.1093/nsr/nwac211

**Published:** 2022-11-02

**Authors:** Luming Li

**Affiliations:** Luming Li National Engineering Research Center of Neuromodulation, Tsinghua University, China

Entering the third decade of the twenty-first century, human society has once again reached a crossroads, and the desire for a new technological revolution has also meant that attention on the brain now goes beyond academia and has become a topic for the whole of society.

Brain–computer interface technology is the focus of our attention. Because it achieves the ‘magical reading from the brain’ and then ‘writing into the brain’, in the limited human cognition of the brain, thinking goes from ‘reading’ to ‘writing’ and then extends to making the brain smarter, and even making the brain ‘immortal’. These scientific imaginations are associated with thinking about oneself and even the ultimate fate of mankind. Research progress in this field also provides the space for wild imagination. It has attracted more commercial investment to enter, which in turn has become the focus of public and social attention. Objectively, this has driven the development of this field, especially the influx of capital. On the other hand, the complexity of the brain is not only driven by capital to make rapid breakthroughs, but requires researchers to think deeply, study real problems and promote the understanding of the brain step by step.

The special topic took this background into account and added the editor's thinking. Differently from the traditional understanding of the brain–computer interface, it focuses on some research progress in the brain–computer interface in a broad sense. Sensing and modulating the human deep brain could help us to understand and treat neurological disorders. The subject of the first review article is different from the conventional information retrieval from the cerebral cortex, whether it is invasive or non-invasive, and focuses on the interaction with the human deep brain. Starting from deep brain stimulation (DBS), it focuses on stimulation to recording, from deep local areas of the brain to the entire subcortical information recording and regulation, which naturally transitions to stereotactic electroencephalography (sEEG) technology, which has recently been widely used in epilepsy clinics. The other review article focuses on optical interface technologies for neuromodulation. Optical neuromodulation through optogenetics is widely used in neuroscience research. Optical interfaces have shown great potential for clinical application in the near future. These are new opportunities brought about by brain–computer interfaces.

This special topic selects four works in perspective and brief communication that represent some of the latest progress, all from highly influential teams. Miguel Nicolelis, who designed the brain interface technology for the disabled to realize the first kick-off for the World Cup in Brazil, considers bidirectional brain–machine interfaces as the foundation for the next generation of neuroprostheses. John Rogers, a pioneer of flexible electronics, covers flexible, stretchable and morphable interface technologies. Grégoire Courtine looks into the possibility of linking the brain and spinal cord together, which may allow paralysed patients to move better through spinal cord epidural electrical stimulation. Yue Chen *et al.* report on the latest progress in DBS as a brain interface. The selection of these pieces of work represents some of the editor's thoughts and expectations in this field: how to promote the study of brain–computer interfaces to serve people, especially those who need it.

The brain–computer interface is a very rapidly developing field. New technologies and new methods are changing with each passing day. Keep paying attention to the latest progress, remain in awe of nature and ethics, think about the connotation of the brain–computer interface and truly promote research on the brain. It will pave a new way for the future of mankind.

## Notes

Guest Editor of Special Topic

